# Association between body mass index and respiratory symptoms in US adults: a national cross-sectional study

**DOI:** 10.1038/s41598-024-51637-z

**Published:** 2024-01-10

**Authors:** Yuefeng Sun, Yueyang Zhang, Xiangyang Liu, Yingying Liu, Fan Wu, Xue Liu

**Affiliations:** 1 The First Clinical Medical College, Shandong University of Traditional Chinese Medicine, Jinan, China; 2 School of Traditional Chinese Medicine, Shandong University of Traditional Chinese Medicine, Jinan, China; 3 Department of Pulmonary and Critical Care Medicine, Affiliated Hospital of Shandong University of Traditional Chinese Medicine, Jinan, China

**Keywords:** Computational biology and bioinformatics, Health care, Medical research, Diseases, Respiratory tract diseases

## Abstract

The correlation between body mass index (BMI) and the development of cough, shortness of breath, and dyspnea is unclear. Therefore, this study aimed to investigate the association between these parameters. Data from individuals who participated in the National Health and Nutrition Examination Survey between 2003 and 2012 were analyzed. Weighted logistic regression analysis and smoothed curve fitting were used to examine the correlation between BMI and respiratory symptoms. In addition, the relationship between BMI, chronic obstructive pulmonary disease (COPD), and bronchial asthma was examined. Stratified analysis was used to discover inflection points and specific groups. Weighted logistic regression and smoothed curve fitting revealed a U-shaped relationship between BMI and respiratory symptoms. The U-shaped relationship in BMI was also observed in patients with bronchial asthma and COPD. Stratified analysis showed that the correlation between BMI and wheezing and dyspnea was influenced by race. In addition, non-Hispanic black individuals had a higher risk of developing cough than individuals of the other three races [OR 1.040 (1.021, 1.060), *p* < 0.0001], and they also exhibited an inverted U-shaped relationship between BMI and bronchial asthma. However, the association of BMI with cough, wheezing, dyspnea, COPD, and asthma was not affected by sex. High or low BMI was associated with cough, shortness of breath, and dyspnea, and has been linked to bronchial asthma and COPD. These findings provide new insights into the management of respiratory symptoms and respiratory diseases.

## Introduction

Respiratory diseases are major contributors to global morbidity and mortality, and respiratory symptoms such as cough, wheezing and exertional dyspnoea are recognisable to patients and may be markers of underlying chronic disease and mortality risk, as well as typical symptoms of chronic obstructive pulmonary disease (COPD) and asthma, which have a serious impact on physical and mental health. Furthermore, the prevalence of asthma has continued to rise over the past three decades^1^. Notably, more than half of the world's population has symptoms associated with one or more respiratory diseases^2–4^. In the general population, respiratory symptoms are an important prognostic indicator of all-cause mortality in people aged 45–75 years^5^. Respiratory symptoms and diseases have a profound impact on an individual's health and quality of life.

Body mass index (BMI) is a widely accepted metric for assessing the degree of obesity and overall health of an individual^6^. Weight and height values are used to calculate a relatively objective parameter, the range of which is used as a measure of body weight^7^. The World Health Organization (WHO) has identified a BMI range of 18.5‒24.9 kg/m^2^ as the generally accepted ideal or healthy weight range^8^. However, obesity was defined as a BMI greater than 30 kg/m^2^^9^. BMI is an affordable and easily obtainable metric widely used in clinical settings. Many patients with obesity have respiratory symptoms and diseases. A meta-analysis found a correlation between obesity and asthma and that asthma risk increases with increasing BMI^10^. Furthermore, obesity reportedly leads to increased chronic inflammation in the body^11,12^. The mechanical effects of obesity result in the narrowing of the airway, which increases respiratory resistance and often manifests as dyspnea and wheezing. However, the “obesity paradox” cannot be ignored^13^. There are relatively few studies on the effect of low body mass index on respiratory symptoms, but some studies have shown that, as the degree of airway obstruction in COPD patients continues to worsen, COPD patients can lead to an increase in respiratory muscle oxygen consumption and work due to the decrease in lung compliance and increase in respiratory resistance, which can trigger symptoms such as dyspnoea and shortness of breath, leading to gastrointestinal dysfunction, feeding difficulties, etc., and then malnutrition, which can lead to atrophy of respiratory muscles, endurance and contraction are reduced, which can further aggravate the gas trap and airway obstruction, and the decline of pulmonary function in patients with COPD^14^.

Despite the impact of both obesity and wasting on lung health, there is limited research on the correlation between body mass index (BMI) and respiratory symptoms (cough, wheeze, and dyspnea) in the general US population. With this in mind, our study aimed to explore the relationship between BMI and respiratory symptoms (cough, wheeze, and dyspnoea) using a nationally representative sample from the National Health and Nutrition Examination Survey (NHANES), and to further investigate the correlation between chronic lung diseases (asthma, COPD), which are typified by respiratory symptoms, and depression (including modifiable factors).

## Materials and methods

### Study population

The National Health and Nutrition Examination Survey (NHANES) is a population-based national cross-sectional survey published by the NCHS^15^. The data collection methods used in this study were approved by the National Center for Health Statistics (NCHS) Ethics Review Board, and written consent was obtained from each participant. Details on Institutional Review Boards of the NCHS are available at (http://www.cdc.gov/nchs/nhanes/irba98.htm). The absence of ethical review was justified by the fact that all data used in the study were publicly accessible and fully anonymized.

The NHANES adopted a complex multistage probability sampling design^16^ to select participants representative of the civilian, non-institutionalized US population. Moreover, the survey is designed to collect information on the health and nutrition of the US household population. Data on the participants’ health, socioeconomic status, and other factors were collected through family interviews. Participants underwent a physical examination at mobile exam centers, which were designed to collect data from physical and laboratory examinations.

**Figure 1:**
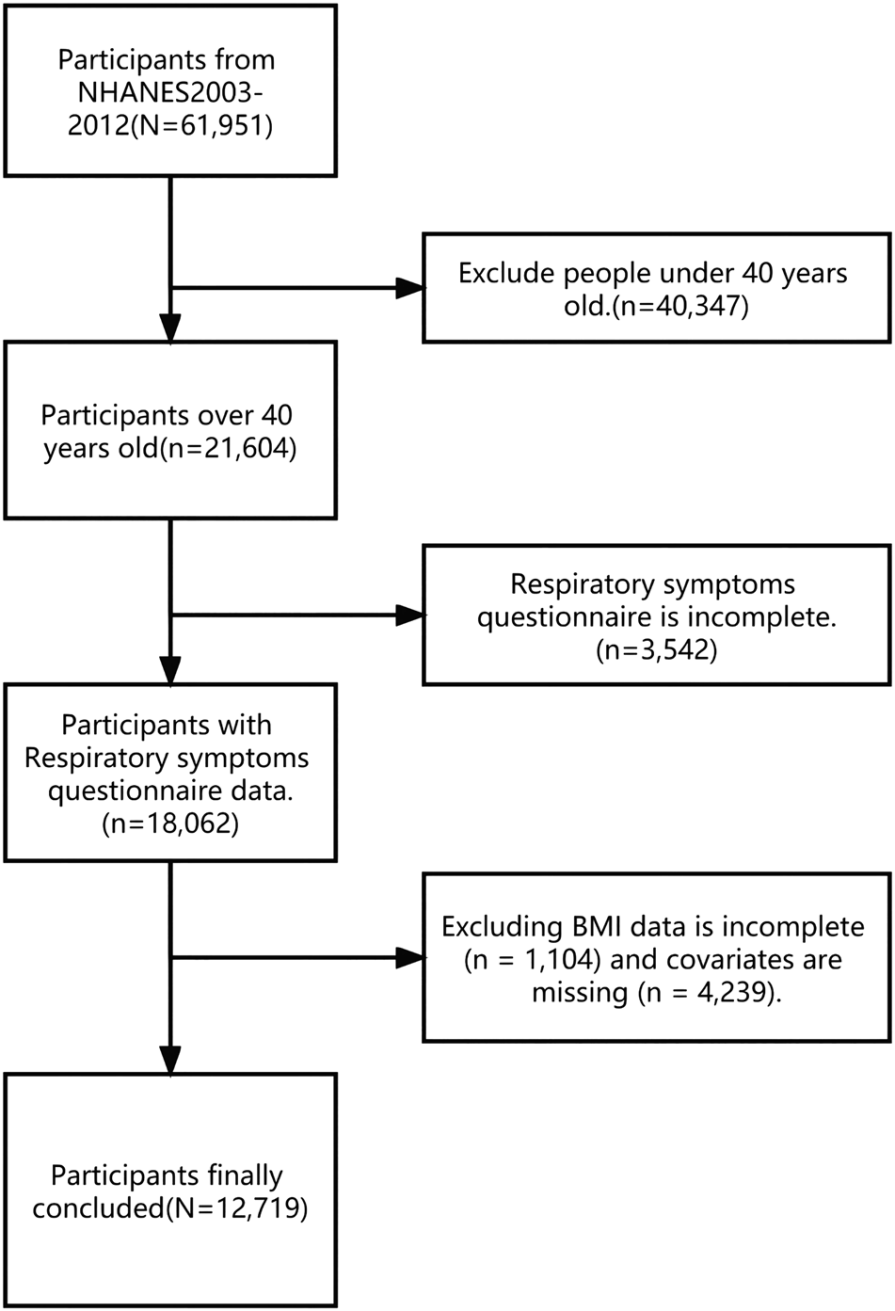
Flow chart of the study population (NHANES 2003–2012).

NHANES data from 2003 to 2012 were used in the study. A total of 61,951 individuals participated in NHANES in 2003‒2012. Participants aged < 40 years (n = 40,347) with incomplete respiratory symptom surveys (n = 3542), incomplete BMI data (n = 1104), and missing covariates (n = 4239) were excluded. Data on poverty-income ratio, marital status, educational attainment, and co-morbidities were included. The study involved 12,719 participants (Fig. 1). The study was conducted in compliance with the RECORD reporting guidelines.

### BMI

Trained health technicians collected BMI data at mobile exam centers and retrieved body measurements from the physical examination module. BMI was calculated as weight (kg)/[height (m)^2^].

### Respiratory symptoms

Coughing was verified with a “yes” answer to the question: “Do you usually cough on most days for 3 consecutive months or more during the year?”.

Wheezing and dyspnea were verified with a “yes” answer to the question: “In the past 12 months, have you had wheezing or whistling in your chest?” and “Do you experience shortness of breath when traveling quickly on level ground or up small inclines?”.

COPD is defined as a diagnosis of “chronic bronchitis or emphysema.” Asthma was verified with a “yes” answer to the question: “Has a doctor or other health professional ever told you that you have asthma?”.

### Study covariates

To mitigate possible bias, we included demographic factors such as age, sex (male/female), race (Mexican–American, non-Hispanic black, non-Hispanic white, other), educational achievement (< high school, high school, college), marital status (divorced/separated/widowed, married/living with a partner, unmarried), household ratio of income to poverty (PIR) (< 1.3, 1.3‒3.5, > 3.5), and smoking status, with intensity measured by the number of cigarettes smoked. Smoking was categorized into three groups: never smoked (smoked less than 100-lifetime cigarettes), former smoker (smoked more than 100-lifetime cigarettes but quit), and current smoker.

### Statistical analysis

All data processing and statistical analysis were performed using R (version 4.2.3) and EmpowerStats software (http://www.EmpowerStats.net). Adjustments were made based on mobile exam center weighting to avoid bias in evaluation. Since underweight (BMI < 18.5 kg/m^2^) participants were only 1.2% of the sample size, we categorised all subjects based on quartiles. To compare differences between groups, weighted χ^2^ tests were used for categorical variables, and weighted linear regression models were used for continuous variables. Three weighted multiple logistic regression models were used to evaluate the relationship between BMI and respiratory symptoms, COPD, and asthma. The significance of weighting is mainly to make the sample better reflect the overall characteristics. The data in NHANES is a stratified equal probability random sample, and the totality represented by each stratum is different, so weighting is required.

**Figure 2:**
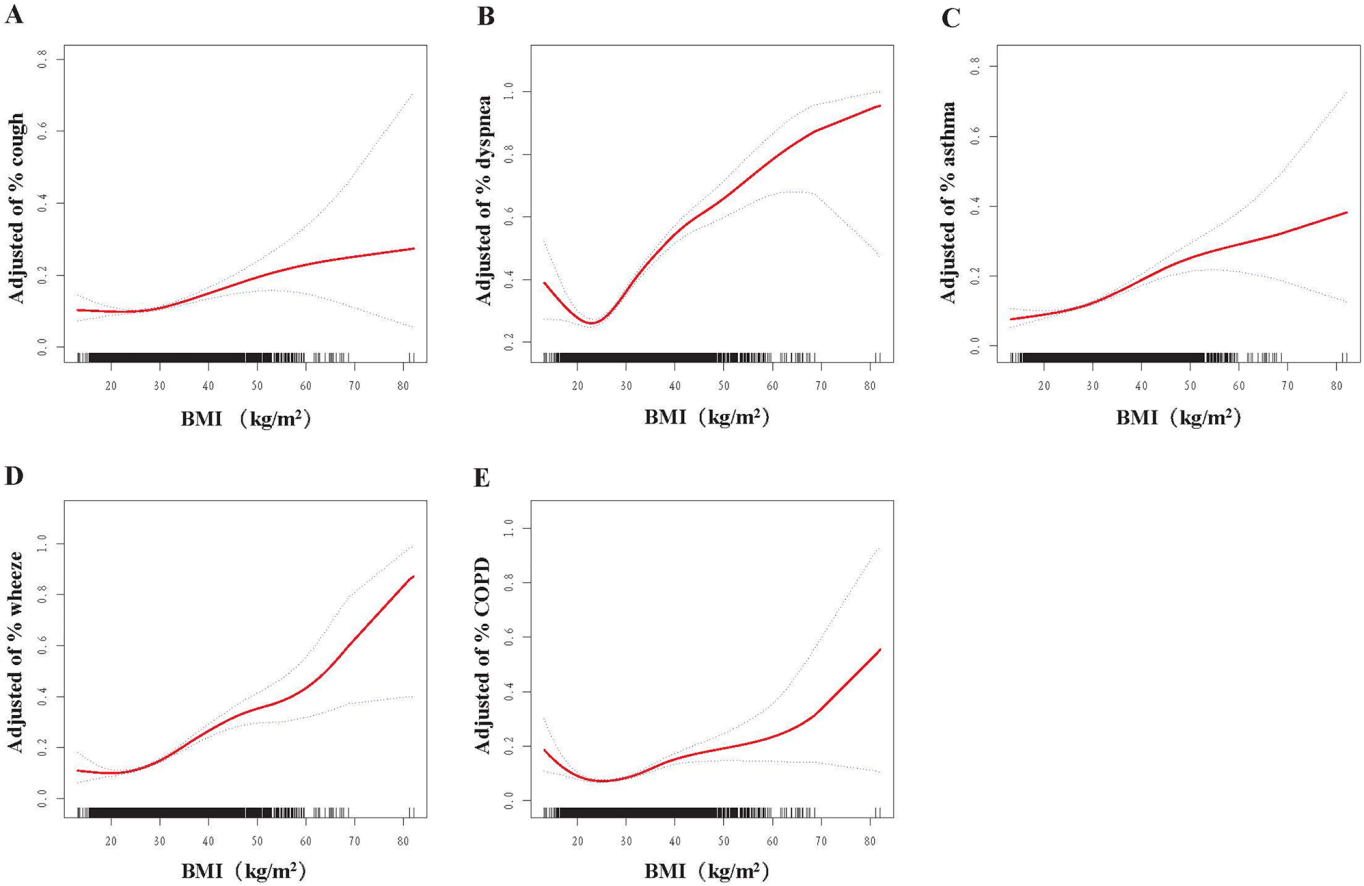
Relationship between BMI and incidence of respiratory symptoms, asthma, and COPD after adjusting for sex, age, race, marital status, PIR, smoking, and education. Panel (**A**) shows the relationship between BMI and the incidence of cough, panel (**B**) shows the relationship between BMI and the incidence of dyspnoea, panel (**C**) shows the relationship between BMI and the incidence of asthma, panel (**D**) shows the relationship between BMI and the incidence of wheezing, and panel (**E**) shows the relationship between BMI and the incidence of chronic obstructive pulmonary disease.

Three models were developed in the study. Model 1 did not account for potential confounding variables. Model 2 adjusted for age, sex, and race. Model 3 adjusted for age, sex, race, marital status, PIR, educational attainment, and smoking. Smoothed curve fitting was used to investigate the possible nonlinear relationships between exposure and outcomes (Fig. 2). If a nonlinear correlation was found, a segmented linear regression model was used to calculate the threshold effect of BMI on respiratory disease based on a smoothing curve. The model was then tested by sex (male, female) and race/ethnicity (non-Hispanic white, Mexican–American, non-Hispanic black, other), and their interaction was also evaluated. Differences with *p* < 0.05 were considered statistically significant.

### Ethical approval

The data collection methods used in this study were approved by the National Center for Health Statistics (NCHS) Ethics Review Board, and written consent was obtained from each participant. Details on Institutional Review Boards of the NCHS are available at (http://www.cdc.gov/nchs/nhanes/irba98.htm). The absence of ethical review was justified by the fact that all data used in the study were publicly accessible and fully anonymized.

## Results

Baseline characteristics of individuals categorized by BMI quartiles are presented in Table 1. Overall, 12,719 participants were included in the study. Of these, 3180 participants had a BMI range of 13.2‒24.9 kg/m^2^, 3175 had a BMI range of 24.9‒28.4 kg/m^2^, 3180 had a BMI range of 28.4‒32.5 kg/m^2^, and 3184 had a BMI range of 32.5‒82 kg/m^2^. Most participants were female (51%) and non-Hispanic white (53.3%), and the prevalence of cough, wheezing, and dyspnea was 11.6%, 10.8%, and 28.2%, respectively. Participants in the lowest BMI quartile (Q1) had higher educational attainment and wealth and comprised a higher proportion of other races than those in the highest BMI quartile (Q4). Table 1 presents the weighted baseline characteristics of applicable participants categorized by BMI quartile. Among the parameters evaluated, smoking was more pronounced in the Q1 group, and the prevalence of smoking-related COPD increased progressively with increasing BMI. The prevalence of respiratory symptoms, COPD, and asthma increased progressively with increasing BMI. In addition, significant differences were observed between the BMI quartile groups with respect to age, race, marital status, annual household income, education, smoking, and the presence of respiratory disease (*p* < 0.05).

**Table 1:** Characteristics of participants: 2003–2012 NHANES (n = 12,719).

Variables	Total, n	BMI (kg/m^2^)				*p* value
	12,719	Q1 13.2‒24.9 (*N* = 3180)	Q2 24.9‒28.4 (*N* = 3175)	Q3 28.4‒32.5 (*N* = 3180)	Q4 32.5‒82 (*N* = 3184)	
Age (year), mean ± SD		60.97 ± 13.52	60.79 ± 12.95	59.91 ± 12.41	58.12 ± 11.46	< 0.001
Sex, n (%)						< 0.001
Female	6485	1744 (54.84%)	1394 (43.91%)	1478 (46.48%)	1869 (58.70%)	
Male	6234	1436 (45.16%)	1781 (56.09%)	1702 (53.52%)	1315 (41.30%)	
Race/ethnicity, n (%)						< 0.001
Mexican American	1880	334 (10.50%)	509 (16.03%)	579 (18.21%)	458 (14.38%)	
Other race	1498	448 (14.09%)	391 (12.31%)	354 (11.13%)	305 (9.58%)	
Non-Hispanic White	6787	1880 (59.12%)	1730 (54.49%)	1619 (50.91%)	1558 (48.93%)	
Non-Hispanic Black	2554	518 (16.29%)	545 (17.17%)	628 (19.75%)	863 (27.10%)	
Marital status, n (%)						< 0.001
Married/living with a partner	8031	1991 (62.61%)	2058 (64.82%)	2094 (65.85%)	1888 (59.30%)	
Divorced/separated/widowed	3779	972 (30.57%)	900 (28.35%)	898 (28.24%)	1009 (31.69%)	
Never married	909	217 (6.82%)	217 (6.83%)	188 (5.91%)	287 (9.01%)	
Poverty, n (%)						< 0.001
< 1.3	3482	841 (26.45%)	800 (25.20%)	844 (26.54%)	943 (29.62%)	
1.3–3.5	4886	1184 (37.23%)	1239 (39.02%)	1221 (38.40%)	1222 (38.38%)	
≥ 3.5	4425	1155 (36.32%)	1136 (35.78%)	1115 (35.06%)	1019 (32.00%)	
Education level, n (%)						< 0.001
< High school diploma	3562	835 (26.26%)	919 (28.94%)	898 (28.24%)	910 (28.58%)	
Completed high school	3024	737 (23.18%)	719 (22.65%)	765 (24.06%)	803 (25.22%)	
≥ College	6133	1608 (50.57%)	1537 (48.41%)	1517 (47.70%)	1471 (46.20%)	
Smoke, n (%)						< 0.001
Never smoker	4765	1463 (46.01%)	1534 (48.31%)	1598 (50.25%)	1633 (51.29%)	
Former smoker	4142	880 (27.67%)	1100 (34.65%)	1088 (34.21%)	1074 (33.73%)	
Current smoker	2349	837 (26.32%)	541 (17.04%)	494 (15.53%)	477 (14.98%)	
Comorbidities, n (%)						
COPD	1148	278 (8.74%)	234 (7.37%)	243 (7.64%)	393 (12.34%)	< 0.001
Bronchial asthma	1560	307 (9.65%)	331 (10.43%)	354 (11.13%)	568 (17.84%)	< 0.001
Respiratory symptoms, n (%)						
Cough	1414	382 (12.01%)	310 (9.76%)	315 (9.91%)	407 (12.78%)	< 0.001
Wheezing	1867	359 (11.29%)	369 (11.62%)	431 (13.55%)	708 (22.24%)	< 0.001
Dyspnea	4516	921 (28.96%)	924 (29.10%)	1116 (35.09%)	1555 (48.84%)	< 0.001

Mean + SD for continuous variables. % for categorical variables. *BMI* body mass index, *COPD* chronic obstructive pulmonary disease.

### Correlation between respiratory symptoms and BMI

Multifactorial logistic regression analysis showed that BMI was positively associated with wheezing and dyspnoea in the unadjusted model [OR 1.053 (1.042, 1.065), OR 1.066 (1.056, 1.076)] (Table 2). These positive correlations persisted after model 3 was corrected for confounders, and all were statistically significant [OR 1.020, 95% CI 1.008–1.031, *p* = 0.001; OR 1.060, 95% CI 1.049–1.071, *p* < 0.0001; OR 1.074, 95% CI 1.064–1.084, *p* < 0.0001]. After converting BMI from a continuous variable to a categorical variable (quartiles), we found that BMI was nonlinearly associated with the risk of respiratory symptoms (Table 2). This nonlinear relationship persisted after correcting for confounders (Table 2). Furthermore, the nonlinear relationship persisted in diseases associated with respiratory symptoms (COPD and asthma). Curve fitting revealed nonlinear and U-shaped correlations (Fig. 2).

**Table 2:** Association of BMI and respiratory symptoms, COPD, and bronchial asthma.

	Model 1 OR (95% CI)	*p* value	Model 2 OR (95% CI)	*p* value	Model 3 OR (95% CI)	*p* value
Cough						
BMI (kg/m^2^)	1.009 (0.997,1.021)	0.134	1.010 (0.998,1.022)	0.107	1.020 (1.008,1.031)	0.001
Q1	Ref		Ref		Ref	
Q2	0.820 (0.690,0.974)	0.025	0.806 (0.684,0.951)	0.011	0.931 (0.787,1.103)	0.404
Q3	0.782 (0.635,0.962)	0.021	0.774 (0.632,0.949)	0.014	0.929 (0.759,1.136)	0.465
Q4	1.083 (0.871,1.345)	0.468	1.091 (0.877,1.358)	0.428	1.301 (1.058,1.600)	0.013
Wheezing						
BMI (kg/m^2^)	1.053 (1.042,1.065)	< 0.0001	1.052 (1.041,1.063)	< 0.0001	1.060 (1.049,1.071)	< 0.0001
Q1	Ref		Ref		Ref	
Q2	0.901 (0.744,1.091)	0.281	0.895 (0.735,1.090)	0.264	0.794 (0.651,0.968)	0.023
Q3	1.083 (0.933,1.258)	0.290	1.079 (0.928,1.255)	0.318	1.117 (0.946,1.318)	0.188
Q4	2.078 (1.759,2.456)	< 0.0001	2.030 (1.708,2.413)	< 0.0001	2.122 (1.765,2.550)	< 0.0001
Dyspnea						
BMI (kg/m^2^)	1.066 (1.056,1.076)	< 0.0001	1.069 (1.059,1.080)	< 0.0001	1.074 (1.064,1.084)	< 0.0001
Q1	Ref		Ref		Ref	
Q2	0.922 (0.798,1.067)	0.272	0.867 (0.746,1.007)	0.062	0.814 (0.699,0.948)	0.009
Q3	1.261 (1.120,1.420)	< 0.001	1.288 (1.144,1.450)	< 0.0001	1.315 (1.155,1.498)	< 0.0001
Q4	2.425 (2.099,2.801)	< 0.0001	2.450 (2.108,2.848)	< 0.0001	2.479 (2.122,2.896)	< 0.0001
COPD						
BMI (kg/m^2^)	1.032 (1.020,1.044)	< 0.0001	1.034 (1.022,1.046)	< 0.0001	1.039 (1.028,1.051)	< 0.0001
Q1	Ref		Ref		Ref	
Q2	1.090 (0.845,1.406)	0.503	1.048 (0.810,1.357)	0.717	0.946 (0.735,1.218)	0.664
Q3	0.995 (0.767,1.290)	0.967	1.012 (0.776,1.319)	0.931	1.038 (0.792,1.361)	0.782
Q4	1.782 (1.462,2.170)	< 0.0001	1.802 (1.462,2.219)	< 0.0001	1.827 (1.477,2.261)	< 0.0001
Bronchial asthma						
BMI (kg/m^2^)	1.046 (1.034,1.058)	< 0.0001	1.043 (1.032,1.055)	< 0.0001	1.044 (1.033,1.056)	< 0.0001
Q1	Ref		Ref		Ref	
Q2	0.899 (0.725,1.115)	0.329	0.836 (0.670,1.043)	0.110	0.819 (0.657,1.019)	0.073
Q3	1.013 (0.820,1.250)	0.905	1.009 (0.816,1.247)	0.936	1.013 (0.819,1.253)	0.903
Q4	1.792 (1.470,2.183)	< 0.0001	1.671 (1.360,2.054)	< 0.0001	1.677 (1.362,2.065)	< 0.0001

Model 1 was unadjusted; model 2 was adjusted for age, sex, race/ethnicity; and model 3 was adjusted for model 2 plus marital, PIR, education, and smoke.

### Subgroup analysis

Because BMI standards vary across sex and race, we conducted stratified analysis by sex and race/ethnicity. The interaction factor of race in wheezing and dyspnea (Table 3) indicates that the correlation between BMI and wheezing and dyspnea is influenced by race. In addition, non-Hispanic black individuals had a higher risk of cough for every 1 kg/m^2^ increase in BMI than the other three races [OR 1.040 (1.021, 1.060), *p* < 0.0001]. However, we did not find an interaction between BMI and respiratory symptoms, COPD, and asthma, implying that their correlation was not affected by gender (Table 4).

**Table 3:** Relationship between body mass index and respiratory symptoms, COPD, and asthma by race.

	Model 1 OR (95% CI)	p for interaction	Model 2 OR (95% CI)	p for interaction	Model 3 OR (95% CI)	p for interaction
Cough		0.762		0.889		0.903
Mexican American	1.020 (0.980,1.062)		1.023 (0.983,1.065)		1.025 (0.987,1.065)	
Other race	1.018 (0.972,1.066)		1.021 (0.975,1.070)		1.023 (0.975,1.073)	
Non-Hispanic White	1.008 (0.994,1.022)		1.008 (0.994,1.023)		1.018 (1.004,1.033)	
Non-Hispanic Black	1.031 (1.014,1.049)		1.030 (1.012,1.049)		1.040 (1.021,1.060)	
Wheezing		0.032		0.024		0.023
Mexican American	1.079 (1.046,1.114)		1.084 (1.052,1.117)		1.085 (1.054,1.117)	
Other race	1.092 (1.055,1.130)		1.091 (1.055,1.128)		1.099 (1.063,1.137)	
Non-Hispanic White	1.050 (1.036,1.064)		1.049 (1.035,1.063)		1.058 (1.044,1.072)	
Non-Hispanic Black	1.053 (1.036,1.069)		1.053 (1.035,1.071)		1.060 (1.041,1.079)	
Dyspnea		0.031		0.041		0.049
Mexican American	1.076 (1.053,1.099)		1.071 (1.048,1.094)		1.073 (1.051,1.096)	
Other race	1.080 (1.050,1.110)		1.083 (1.054,1.112)		1.083 (1.054,1.113)	
Non-Hispanic White	1.067 (1.055,1.079)		1.072 (1.059,1.085)		1.077 (1.064,1.090)	
Non-Hispanic Black	1.055 (1.043,1.068)		1.051 (1.037,1.064)		1.058 (1.044,1.072)	
COPD		0.321		0.388		0.387
Mexican American	1.051 (0.999,1.104)		1.049 (0.997,1.103)		1.049 (1.001,1.100)	
Other race	1.056 (1.003,1.111)		1.060 (1.006,1.118)		1.066 (1.015,1.119)	
Non-Hispanic White	1.030 (1.016,1.044)		1.033 (1.019,1.047)		1.039 (1.026,1.052)	
Non-Hispanic Black	1.042 (1.023,1.061)		1.037 (1.019,1.056)		1.042 (1.024,1.060)	
Bronchial Asthm**a**		0.214		0.094		0.079
Mexican American	1.055 (1.017,1.094)		1.048 (1.012,1.085)		1.046 (1.010,1.082)	
Other race	1.054 (1.015,1.094)		1.053 (1.016,1.092)		1.051 (1.013,1.091)	
Non-Hispanic White	1.047 (1.032,1.062)		1.046 (1.032,1.060)		1.047 (1.033,1.061)	
Non-Hispanic Black	1.040 (1.024,1.056)		1.039 (1.022,1.055)		1.040 (1.023,1.056)	

Model 1 was unadjusted; model 2 was adjusted for age, sex; and model 3 was adjusted for model 2 plus marital, PIR, education, and smoke.

*Stratified variables themselves were also not adjusted in the subgroup analysis.

**Table 4:** Relationship between body mass index and respiratory symptoms, COPD, and asthma by sex.

	Model 1 OR (95% CI)	*p* for interaction	Model 2 OR (95% CI)	*p* for interaction	Model 3 OR (95% CI)	p for interaction
Cough		0.022		0.026		**0.07**
Male	0.990 (0.968,1.012)		0.992 (0.970,1.014)		1.007 (0.988,1.025)	
Female	1.019 (1.005,1.033)		1.019 (1.005,1.033)		1.027 (1.012,1.042)	
Wheezing		0.507		0.524		0.96
Male	1.049 (1.033,1.066)		1.048 (1.031,1.065)		1.060 (1.043,1.076)	
Female	1.055 (1.043,1.068)		1.054 (1.041,1.066)		1.060 (1.046,1.074)	
Dyspnea		0.578		0.744		0.564
Male	1.063 (1.048,1.079)		1.068 (1.053,1.084)		1.081 (1.067,1.096)	
Female	1.068 (1.057,1.079)		1.070 (1.059,1.081)		1.071 (1.059,1.083)	
COPD		0.413		0.341		0.129
Male	1.039 (1.017,1.061)		1.047 (1.024,1.071)		1.058 (1.034,1.082)	
Female	1.028 (1.014,1.042)		1.028 (1.014,1.042)		1.030 (1.016,1.044)	
Bronchial Asthma		0.961		0.936		0.745
Male	1.045 (1.026,1.065)		1.044 (1.025,1.064)		1.046 (1.027,1.066)	
Female	1.045 (1.032,1.058)		1.043 (1.030,1.057)		1.044 (1.030,1.058)	

Model 1 was unadjusted; model 2 was adjusted for age, race/ethnicity; and model 3 was adjusted for model 2 plus marital, PIR, education, and smoke.

*Stratified variables themselves were also not adjusted in the subgroup analysis.

### Smoothing curve fitting analysis

**Figure 3:**
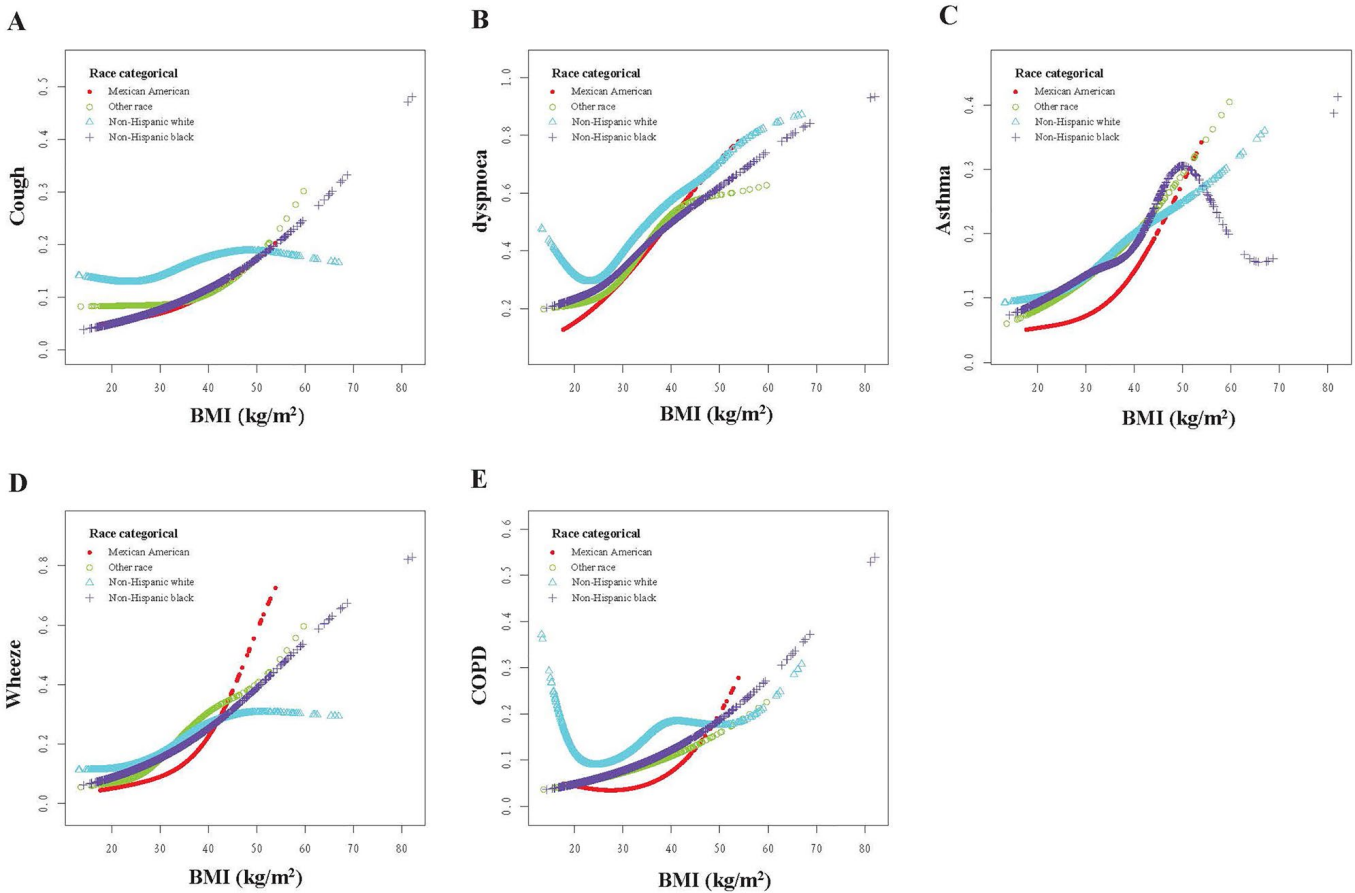
The relationship between BMI and the incidence of respiratory symptoms, asthma, and COPD after adjusting for sex, age, marital status, income, smoking, and education. Panel (**A**) shows the relationship between BMI and the incidence of cough, panel (**B**) shows the relationship between BMI and the incidence of dyspnoea, panel (**C**) shows the relationship between BMI and the incidence of asthma, panel (**D**) shows the relationship between BMI and the incidence of wheezing, and panel (**E**) shows the relationship between BMI and the incidence of COPD.

After adjusting for all covariates (Model 3), smoothing curves were used to visualize the relationship between BMI and respiratory symptoms, COPD, and asthma across races (Fig. 3). The overall smoothing curve indicates a nonlinear relationship between BMI and respiratory symptoms, with inflection points of 25.56 kg/m^2^, 20.85 kg/m^2^, and 22.7 kg/m^2^ (log-likelihood ratio = 0.006; log-likelihood ratio = 0.002; log-likelihood ratio < 0.001). The same U-shaped relationship was found in COPD, with an inflection point of 24.12 kg/m^2^ (log-likelihood ratio < 0.001) (Fig. 3; Table 5). Some U-shaped relationships were also found when the results were analyzed by race, with non-Hispanic white individuals having an optimal BMI of 22.68 kg/m^2^ [OR 0.899 (0.847, 0.955), *p* = 0.0005; OR 1.076 (1.066, 1.086), *p* < 0.0001] associated with the lowest odd ratio (OR) for dyspnea symptoms, and Mexican–American and non-Hispanic white individuals having optimal BMIs of 26.91 kg/m^2^ [OR 0.850 (0.742, 0.974) *p* = 0.0191; OR 1.098 (1.048, 1.149) *p* < 0.0001) and 22.21 kg/m^2^ (OR 0.809 (0.744, 0.880) *p* < 0.0001; OR 1.043 (1.030, 1.056) *p* < 0.0001] associated with the lowest OR for COPD prevalence, respectively. However, a significant nonlinear relationship was observed between BMI and COPD (log likelihood ratio = 0.046) among non-Hispanic black individuals, the probability of COPD prevalence increased for each BMI unit change (> 24.85 kg/m^2^) (OR 1.053 (1.033, 1.073) *p* < 0.0001) (Table 5). In addition, among non-Hispanic black individuals, there was an inverted U-shaped relationship between BMI and bronchial asthma (Fig. 3); however, this relationship was not described in Table 5, which may be related to differences in analysis methods or insufficient sample size.

**Table 5:** Threshold analysis between BMI and respiratory tract symptoms, COPD, and asthma.

Stratified by race	Total	Mexican American	Other race	Non-Hispanic White	Non-Hispanic Black
	OR (95% CI)* p* value	OR (95% CI)* p* value	OR (95% CI)* p* value	OR (95% CI)* p* value	OR (95% CI)* p* value
Cough					
Standard logistic model	1.025 (1.016, 1.034) < 0.0001	1.028 (0.995, 1.063) 0.0968	1.018 (0.987, 1.050) 0.2532	1.017 (1.006, 1.029) 0.0029	1.043 (1.024, 1.062) < 0.0001
Inflection point (K)	25.65	30.56	36.6	20.46	22.55
BMI < K	0.977 (0.944, 1.011) 0.1829	0.973 (0.909, 1.041) 0.4220	0.989 (0.949, 1.030) 0.5810	0.882 (0.766, 1.017) 0.0835	0.922 (0.782, 1.086) 0.3291
BMI > K	1.034 (1.023, 1.045) < 0.0001	1.068 (1.015, 1.123) 0.0108	1.102 (1.022, 1.189) 0.0112	1.021 (1.009, 1.033) 0.0006	1.048 (1.028, 1.067) < 0.0001
Log-likelihood ratio	0.006	0.074	0.039	0.055	0.158
Wheezing					
Standard logistic model	1.062 (1.055, 1.070) < 0.0001	1.104 (1.074, 1.135) < 0.0001	1.097 (1.071, 1.124) < 0.0001	1.051 (1.041, 1.062) < 0.0001	1.064 (1.049, 1.079) < 0.0001
Inflection point (K)	20.85	34.27	37.89	39.82	21.78
BMI < K	0.893 (0.804, 0.993) 0.0358	1.047 (0.999, 1.098) 0.0547	1.117 (1.080, 1.156) < 0.0001	1.063 (1.049, 1.076) < 0.0001	0.957 (0.798, 1.146) 0.6322
BMI > K	1.066 (1.058, 1.074) < 0.0001	1.176 (1.113, 1.243) < 0.0001	1.041 (0.970, 1.118) 0.2623	1.006 (0.970, 1.043) 0.7475	1.066 (1.051, 1.082) < 0.0001
Log-likelihood ratio	0.002	0.009	0.121	0.010	0.268
Dyspnea					
Standard logistic model	1.065 (1.058, 1.071) < 0.0001	1.086 (1.065, 1.108) < 0.0001	1.073 (1.052, 1.095) < 0.0001	1.063 (1.054, 1.072) < 0.0001	1.057 (1.044, 1.069) < 0.0001
Inflection point (K)	22.7	22.6	20.88	22.68	23.04
BMI < K	0.921 (0.878, 0.966) 0.0007	0.945 (0.741, 1.206) 0.6517	0.871 (0.671, 1.130) 0.2975	0.899 (0.847, 0.955) 0.0005	0.968 (0.875, 1.071) 0.5258
BMI > K	1.074 (1.067, 1.081) < 0.0001	1.090 (1.068, 1.113) < 0.0001	1.078 (1.056, 1.101) < 0.0001	1.076 (1.066, 1.086) < 0.0001	1.061 (1.048, 1.075) < 0.0001
Log-likelihood ratio	< 0.001	0.279	0.130	< 0.001	0.091
COPD					
Standard logistic model	1.036 (1.027, 1.046) < 0.0001	1.052 (1.011, 1.095) 0.0120	1.057 (1.024, 1.091) 0.0006	1.028 (1.016, 1.040) < 0.0001	1.044 (1.026, 1.062) < 0.0001
Inflection point (K)	24.12	26.91	23.96	22.21	24.85
BMI < K	0.899 (0.857, 0.942) < 0.0001	0.850 (0.742, 0.974) 0.0191	1.112 (0.887, 1.395) 0.3558	0.809 (0.744, 0.880) < 0.0001	0.932 (0.839, 1.037) 0.1960
BMI > K	1.052 (1.041, 1.062) < 0.0001	1.098 (1.048, 1.149) < 0.0001	1.053 (1.016, 1.091) 0.0050	1.043 (1.030, 1.056) < 0.0001	1.053 (1.033, 1.073) < 0.0001
Log-likelihood ratio	< 0.001	0.004	0.646	< 0.001	0.046
Bronchial Asthma					
Standard logistic model	1.044 (1.036, 1.052) < 0.0001	1.061 (1.030, 1.092) < 0.0001	1.052 (1.027, 1.078) < 0.0001	1.043 (1.032, 1.054) < 0.0001	1.043 (1.028, 1.058) < 0.0001
Inflection point (K)	21.81	27.3	21.2	21.58	44.82
BMI < K	0.971 (0.887, 1.063) 0.5274	0.957 (0.855, 1.070) 0.4371	1.887 (0.862, 4.130) 0.1119	0.928 (0.830, 1.039) 0.1957	1.052 (1.033, 1.071) < 0.0001
BMI > K	1.046 (1.038, 1.055) < 0.0001	1.082 (1.044, 1.122) < 0.0001	1.045 (1.018, 1.072) 0.0008	1.047 (1.036, 1.059) < 0.0001	1.011 (0.965, 1.058) 0.6515
Log-likelihood ratio	0.127	0.075	0.056	0.051	0.149

Variables were adjusted for age, sex, marital status, race/ethnicity, PIR, education and smoke.

## Discussion

In this cross-sectional study of Americans aged > 40 years, a U-shaped relationship between BMI and respiratory symptoms was observed. A U-shaped relationship between BMI and all-cause mortality, including respiratory diseases, has been reported previously^17^. Thus, our study supports similar previous findings with a different population sample. According to our findings, a U-shaped correlation exists between BMI and cough, wheezing, and dyspnea. In addition to respiratory symptoms, the association between BMI and asthma and COPD was found to follow a U-shaped pattern. The risk of respiratory symptoms, COPD, and bronchial asthma increased when BMI was less than or greater than the inflection point.

Cough, wheezing, and dyspnea are the most common respiratory symptoms and are common in patients with COPD and asthma. Respiratory symptoms are an important indicator of various respiratory diseases, which increase the risk of death due to pulmonary disease, particularly obstructive pulmonary disease^18^. The Global Lung Initiative defines wheezing and dyspnea as predictors of poor prognosis in pulmonary dysfunction, and cough is associated with a poor prognosis in COPD^19,20^. BMI is an accepted indicator of health and obesity, and coughing, wheezing, and shortness of breath are associated with cardiopulmonary disease. Dyspnea and wheezing are common in people with high BMI^21,22^. BMI ≥ 30 kg/m^2^ is one of the diagnostic criteria for obesity hypoventilation syndrome, of which dyspnea is a typical clinical symptom. Large fat deposits also alter the mechanical properties of respiration, leading to wheezing^23,24^. Furthermore, BMI is negatively correlated with blood oxygen levels and significantly affects lung capacity in obese individuals with BMI ≥ 30 kg/m^2^, even if they do not have cardiopulmonary disease^25^. Landt et al*.* found a correlation between BMI and chronic cough, indicating that individuals with obesity are two to three times more likely to develop cough than healthy individuals^26^.

The U-shaped correlation between BMI and COPD and asthma suggests that high or low BMI can negatively affect the respiratory system. Lambert et al*.* found that obese patients with COPD have a poor prognosis, including increased dyspnea and activity limitation, which severely affects their quality of life^27^. Weight loss is often recommended for people with high BMI. However, the obesity paradox states that high BMI sometimes has a differential effect on COPD morbidity and mortality; excessive weight loss can increase COPD mortality^28^. Thus, whether patients with COPD should be encouraged to lose weight remains unclear.

We stratified analyses by race to examine differences in the effect of BMI on respiratory symptoms between races. Notably, some differences in BMI standards exist between different races; however, no specific numerical classification standards exist. It is generally believed that the body fat rate of black individuals is lower compared to other races. If calculated according to the BMI formula, black individuals may have a higher BMI than the standard values; however, owing to differences in socioeconomic status, the prevalence of obesity in black individuals is also higher^29^.

Obesity-related health disparities are particularly prevalent among Mexican Americans^30^. Among US adults aged ≥ 20 years, being overweight or obese is more common among Mexican Americans^31^. The prevalence of obesity and obesity-related complications is exacerbated in the U.S.-Mexico border region^32^. Black individuals have higher bone mass and bone density than white individuals, which contributes to the generally higher BMI in black individuals and explains the higher optimal BMI associated with the lowest risk of COPD prevalence among Mexican-Americans and non-Hispanic black individuals compared with white individuals in our study^33^.

The U-shaped relationship between BMI and respiratory symptoms suggests that attention should not be focused only on those with a high BMI; too low a BMI can also be detrimental to health. Studies have shown that too low a BMI increases the risk of cardiovascular disease, and a phenotype with a BMI of less than 18.5 kg/m^2^ is associated with an increased risk of all-cause mortality^34,35^. Low BMI increases the risk of death from respiratory diseases^36,37^. Individuals with lower BMI are more likely to develop COPD and have lower lung function compared to those with higher BMI^38^. These results suggest that the risk of small airway obstruction in underweight individuals deserves more attention and that excessive wasting may also affect the prognosis of patients with COPD.

Our study differs from previous studies in that a nationally representative sample that includes potential covariates was used, which makes our findings more generalizable and reliable. Nevertheless, some limitations must be considered in the interpretation of these results. Since the NHANES database is a cross-sectional database, it was impossible to distinguish between cause and effect. Second, despite the statistical techniques used to adjust for confounding variables, the possibility of interference from other confounding factors cannot be completely ruled out (Supplementary Information 1).

## Conclusion

We demonstrated that both excessively high and excessively low BMIs are associated with respiratory symptoms, such as cough, asthma, and dyspnea, as well as between asthma and COPD, which exhibit U-shaped relationships. These observations may help us better understand and manage respiratory diseases.

### Supplementary Information

Supplementary Information 1.

Supplementary Information 2.

## Data Availability

The raw data supporting the conclusions of this article will be made available by the authors, without undue reservation. All relevant data are within its Supporting Information files. The datasets used in this study are freely available from the National Health and Nutrition Examination Survey (NHANES). The data can be accessed at: https://www.cdc.gov/nchs/nhanes/index.htm.
